# Dissemination of *bla*_NDM-5_ gene via an IncX3-type plasmid among non-clonal *Escherichia coli* in China

**DOI:** 10.1186/s13756-018-0349-6

**Published:** 2018-04-26

**Authors:** Xi Li, Ying Fu, Mengyuan Shen, Danyan Huang, Xiaoxing Du, Qingfeng Hu, Yonglie Zhou, Dairong Wang, Yunsong Yu

**Affiliations:** 1 Centre of Laboratory Medicine, Zhejiang Provincial People’s Hospital, People’s Hospital of Hangzhou Medical College, 158 Shangtang Road, Zhejiang, 310014 Hangzhou China; 2 Department of Clinical Laboratory, Sir Run Run Shaw Hospital, College of Medicine, Zhejiang University, Zhejiang, 310016 Hangzhou China; 3 Department of Infectious Diseases, Sir Run Run Shaw Hospital, College of Medicine, Zhejiang University, Zhejiang, 310016 Hangzhou China; 4 Blood Center of Zhejiang Province, 789 Jianye Road, Zhejiang, 310052 Hangzhou China

**Keywords:** *Enterobacteriaceae*, Carbapenem resistance, *bla*_NDM-5_, IncX3 type plasmid

## Abstract

**Background:**

The emergence and spread of New Delhi metallo-β-lactamase-producing *Enterobacteriaceae* has been a serious challenge to manage in the clinic due to its rapid dissemination of multi-drug resistance worldwide. As one main type of carbapenemases, New Delhi metallo-β-lactamase (NDM)is able to confer resistance to almost all β-lactams, including carbapenems, in *Enterobacteriaceae*. Recently, New Delhi metallo-β-lactamase-5 attracted extensive attention because of increased resistance to carbapenems and widespread dissemination. However, the dissemination mechanism of *bla*_NDM-5_ gene remains unclear.

**Methods:**

A total of 224 carbapenem-resistant *Enterobacteriaceae* isolates (CRE) were collected from different hospitals in Zhejiang province. NDM-5-positive isolates were identified and subjected to genotyping, susceptibility testing, and clinical data analysis. We established the genetic location of *bla*_NDM-5_ with southern blot hybridisation, and analysed plasmids containing *bla*_NDM-5_ with filter mating and DNA sequencing.

**Results:**

Eleven New Delhi metallo-β-lactamase-5 (NDM-5)-producing strains were identified, including 9 *Escherichia coli* strains, 1 *Klebsiella pneumoniae* strain, and *1 Citrobacter freundii* strain. No epidemiological links for *E. coli* isolates were identified by multilocus sequence typing (MLST) and pulsed-field gel electrophoresis (PFGE). S1-PFGE and southern blot suggested that the *bla*_NDM-5_ gene was located on a 46-kb IncX3-type plasmid in all isolates. Nine of the 11 isolates (81.8%) tested could successfully transfer their carbapenem-resistant phenotype to *E. coli* strain C600. Moreover, sequence analysis further showed that this plasmid possessed high sequence similarity to most of previously reported *bla*_NDM-5_-habouring plasmids in China.

**Conclusion:**

The present data in this study showed the IncX3 type plasmid played an important role in the dissemination of *bla*_NDM-5_ in *Enterobacteriaceae*. In addition, to the best of our knowledge, this report is the first to isolate both *E. coli* and *C. freundii* strains carrying *bla*_NDM-5_ from one single patient, which further indicated the possibility of *bla*_NDM-5_ transmission among diverse species. Close surveillance is urgently needed to monitor the further dissemination of NDM-5-producing isolates.

**Electronic supplementary material:**

The online version of this article (10.1186/s13756-018-0349-6) contains supplementary material, which is available to authorized users.

## Background

*Enterobacteriaceae*, such as *E.coli*, *K. pneumoniae* and *C. freundii*, are important pathogens that cause human infections. Carbapenem antibiotics are used in the treatment of infections caused by multi-drug resistant *Enterobacteriaceae*. However, the emergence of Carbapenem-resistant *Enterobacteriaceae* (CRE) has been a serious challenge to manage in the clinic because of the rapid worldwide dissemination of multi-drug resistance [1]. As one main type of carbapenemases, New Delhi metallo-β-lactamase (NDM)is able to confer resistance to almost all β-lactams, including carbapenems, in *Enterobacteriaceae*. Since the first report of *bla*_NDM-1_, 17 variants of NDM enzymes (NDM-1 to NDM-17) have been identified among Gram-negative bacteria worldwide (http://www.ncbi.nlm.nih.gov/pathogens/submit_beta_lactamase/). Among NDM carbapenemases, New Delhi metallo-β-lactamase-5, first identified in an *E. coli* strain in the UK in 2011, attracted extensive attention because of increased resistance to carbapenems and broad-spectrum cephalosporins [2]. In addition, *bla*_NDM-5_ was reported to be carried in different incompatibility typing plasmids to transfer [3], such as IncF, IncN and IncX3. These plasmids are able to facilitate the dissemination of *bla*_NDM-5_ among the members of *Enterobacteriaceae* through horizontal gene transfer. NDM-5-producing isolates have been identified worldwide, such as in America [4], Australia [5], China [6], Denmark [7] and India [8]. Furthermore, NDM-5-positive strains were not only isolated from clinical specimens but also from animals, such as dogs [9], cats [10] and cows [11]. Worryingly, *bla*_NDM-5_ has also been identified in environmental samples [hospital sewage water [12] and urban river [13]], indicating its presence in the community. However, the dissemination mechanism of *bla*_NDM-5_ gene remains unclear.

In this study, we screened NDM-5-producing *Enterobacteriaceae* to elucidate the dissemination mechanism. In addition, to the best of our knowledge, this report is the first to isolate *E. coli* and *C. freundii* strains carrying *bla*_NDM-5_ from the same patient.

## Methods

### Bacterial strains

From Jun. 2016 to Sep. 2017, 224 carbapenem-resistant *Enterobacteriaceae* isolates, as determined by the agar dilution method according to the Clinical and Laboratory Standards Institute guidelines [14], were obtained from four hospitals in different locations in Zhejiang, China. In a retrospective study, common carbapenemase genes (*bla*_KPC_, *bla*_IMP_, *bla*_VIM_, *bla*_OXA-48_, and *bla*_NDM_) were amplified, and the positive products were sequenced; eleven NDM-5 producing strains were identified for further study. The NDM-5 producing strains were preliminarily identified by the VITEK 2 system (Sysmex-bioMérieux, Marcy l’Etoile, France) and further confirmed by whole genome sequencing. The characteristics of the isolates and related clinical data are shown in Table 1.

**Table 1:** Clinical characteristics

Isolates	Date of hospitalization	Date of isolation	Patient Sex	Patient Age (years)	Clinical Sample	Hospital Ward	Clinical Diagnosis	Antimicrobial Therapy	Outcome
EC135	2016/5/27	2016/6/20	Male	85	Sputum	ICU	Acute renal failure	CPS, LEV	Death
KP387	2017/6/7	2017/6/26	Male	40	blood	Hematology	Myelodysplastic syndromes	TGC, LEV, AMK	Alive
EC126	2016/7/29	2016/8/10	Female	76	urine	Surgery	Uracratia	CPS, TGC	Alive
EC734	2016/7/27	2016/9/9	Female	61	pus	ICU	Kidney neoplasms	CPS, IMP, LEV, TGC	Death
EC463	2016/10/7	2016/10/24	Male	16	blood	Hematology	Acute lymphoblastic leukemia	AMK, IMP, TZP	Alive
EC144	2016/10/24	2016/11/3	Female	50	ascites	Surgery	Gastric cancer	CPS, AMK	Alive
EC122	2017/5/5	2017/5/23	Male	69	urine	ICU	Aspiration pneumonia	TZP, CPS, LEV	Alive
EC611	2017/6/12	2017/7/5	Male	72	ascites	Surgery	Colonic neoplasms	TZP, CPS, IMP	Alive
EC418	2017/7/11	2017/7/22	Female	27	feces	Hematology	Acute myelogenous leukemia	IMP, MEM, LEV	Alive
CF418	2017/7/11	2017/7/22	Female	27	feces	Hematology	Acute myelogenous leukemia	IMP, MEM, LEV	Alive
EC310	2017/6/20	2017/7/29	Female	55	blood	Infectious Disease	Biliary tract infection	CPS, IMP, LEV, ATM, AMK, TGC	Alive

*MNO* minocycline, *MEM* meropenem, *LEV* levofloxacin, *TZP* piperacillin/tazobactam, *CPS* cefperazone/sulbactam, *TGC* tigecycline, *IMP* imipenem, *AMK* amikacin

### Antimicrobial susceptibility testing

Antimicrobial susceptibility testing was performed using broth microdilution method [14]. The antibiotics tested in this study were amikacin, aztreonam, cefepime, ceftazidime, ciprofloxacin, gentamicin, imipenem, minocycline, colistin and tigecycline. The results were analysed according to the CLSI guidelines [14], except tigecycline and colistin, for which the European Committee on Antimicrobial Susceptibility Testing breakpoints were used (http://www.eucast.org/clinical_breakpoints). *E. coli* ATCC 25922 was used as a quality control strain.

### Bacterial genotyping

Pulsed-field gel electrophoresis (PFGE) was performed to analyse the clonal relatedness of the NDM-5 producing *E. coli* isolates according to the previous study [15]. Briefly, the isolates were digested by *X*baI endonuclease, which was carried out with a CHEF-Mapper XA PFGE system (Bio-Rad, USA) with a 5–35 s linear ramp for 22 h at 6 V/cm and 14 °C. The PFGE profiles were analyzed with BioNumerics software (Applied Maths, Sint-Martens-Latern, Belgium). The *Salmonella enterica* serotype Braenderup H9812 was used as the size marker.

MLST was also performed for molecular typing. Bacterial genomic DNA was extracted from these isolates. Seven housekeeping genes of *E. coli* (*adk*, *fumC*, *gyrB*, *icd*, *mdh*, *purA* and *recA*), and *K. pneumoniae* (*gapa*, *infb*, *mdh*, *pgi*, *phoe*, *rpob*) were amplified by PCR, and the products were sequenced to analyse the ST.

### Southern blot analysis and conjugation experiments

To determine the plasmid location of the *bla*_NDM-5_ gene, genomic DNA digested with S1-nuclease (TaKaRa, Japan) was electrophoresed on a CHEF-mapper XA pulsed-field gel electrophoresis (PFGE) system (Bio-Rad, USA) for 18 h at 14 °C with run conditions of 6 V/cm and pulse times from 2.16 s to 63.8 s. The DNA fragments were transferred to a positive-charged nylon membrane (Millipore, USA) and then hybridized with a digoxigenin-labeled *NDM-5*-specific probe. An NBT/BCIP color detection kit (Roche, Germany) was then used to detect the fragments. The *Salmonella enterica* serotype Braenderup H9812 was used as the size marker.

A filter-mating experiment was performed between the *bla*_NDM-5_-positive isolates and rifampicin-resistant *E. coli* C600 as the recipient strain [15]. Transconjugants were selected on Mueller-Hinton agar plates containing 500 mg/L rifampicin and 100 mg/L ampicillin. PCR sequencing and antimicrobial susceptibility testing of the transconjugants were subsequently carried out to confirm whether the plasmid was successfully transferred to the recipient.

### Plasmids analysis

Plasmid extraction and analysis was performed as previously described [15]. Briefly, the plasmid DNA of strains was extracted using a QIAamp DNA MiniKit (Qiagen, Valencia, CA, USA) following the manufacturer’s recommendations. The plasmids were sequenced on an Illumina-Hiseq™ 2000 (Illumina Inc., San Diego, U.S.A) platform with 2 × 100 bp paired-end reads. Sequence reads were assembled using CLC Genomics Workbench software package (CLC Bio 8.0). Gaps of a representative plasmid were closed by standard PCR and Sanger sequencing according to previous study [16]. The RAST (Rapid Annotation using Subsystems Technology) annotation website server (http://rast.nmpdr.org/rast.cgi) was then used to annotate the genomes of the plasmid. The circular map of the pEC463-NDM5 plasmid was generated using the CGview server [17]. A comparison of pEC463-NDM5 and three related plasmids was performed with EasyFig 2.2.2 [18]. The rested plasmid sequences were mapped to the representative plasmid sequence with CLC genomics workbench version 8.0.

Incompatibility typing of the *bla*_NDM_ plasmid was performed by PCR-based replicon typing [19, 20] and was further identified with the help of PlasmidFinder-1.3 server (https://cge.cbs.dtu.dk/services/PlasmidFinder/).

In addition, plasmid stability was determined [3]. Briefly, the *bla*_NDM-5_-positive isolates were individually streaked out in the MH agar, incubated at 37 °C for 24 h, and then transferred to a fresh MH agar. After repeating this procedure for 12 days, 12 individual colonies were randomly selected. Subsequently, the *bla*_NDM-5_ gene was screened by PCR and sequenced.

### Nucleotide sequence accession number

The complete sequence of the plasmid pEC463-NDM5 (accession number MG545911), is deposited at DDBJ/EMBL/GenBank.

## Results and discussion

### Isolate characteristics and antimicrobial susceptibility testing

Among the 224 CRE isolates, 137 isolates were KPC-2 carbapenemase producers, eleven isolates were NDM-5 carbapenemase producers, four isolates carried *bla*_IMP-1_ gene, two isolates carried *bla*_VIM-1_ gene and two isolates carried *bla*_NDM-1_ gene. In addition, 68 isolates exited other unknown mechanism of carbapenem-resistance.

In this study, eleven NDM-5-producing isolates were further identified, including nine *E. coli*, one *K. pneumoniae* and one *C. freundii*. These isolates were all recovered from hospitalized patients. These patients were aged between 16 and 85 years, with an average age of 55 years, had different severities of illness (Table 1), and all had previously received broad-spectrum antibiotics. Notably, with both *E. coli* (EC418) and *C. freundii* strains (CF418) were isolated from the feces of one patient from haematology department. This patient was found to be a carrier of *bla*_NDM-5_-positive strains. In contrast, the other patients from whom *bla*_NDM-5_-carrying strains were isolated from blood, pus, ascites, urine or sputum were symptomatic. In addition, these patients had no recent history of travel or hospitalization abroad.

**Fig. 1:**
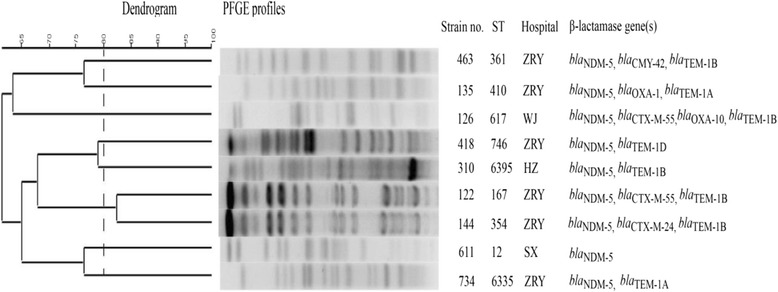
The dendrogram is based on the similarity of PFGE patterns from 9 *bla*NDM-5 positive clinical *E. coli* isolates. The right illustrates results from MLST, hospitals and β-lactamase gene(s)

The antimicrobial susceptibility testing results showed that the *bla*_NDM-5_-positive isolates were resistant to carbapenems, third-generation cephalosporins, and cefperazone/sulbactam. These isolates were also resistant to fluoroquinolones (81.8%), aztreonam (36.4%), amikacin (36.4%), nitrofurantoin (45.4%) and tigecycline (18.2%). All isolates were susceptible to colistin. *E.coli* EC122 and *K. pneumoniae* KP387 strains were both resistant to tigecycline, suggesting that increased resistance phenotypes of *bla*_NDM-5_-postive isolates are increasing in clinics. In addition, other β-lactamase genes, such as those encoding CTX-M-24, CTX-M-55, CMY-42, were also frequently detected in various *bla*_NDM-5_-positive *E. coli* strains (Fig. 1). Gene encoding SHV-1 and CMY-26 were detected in the *K. pneumoniae* KP387 and *C. freundii* CF418 strains, respectively.

**Fig. 2:**
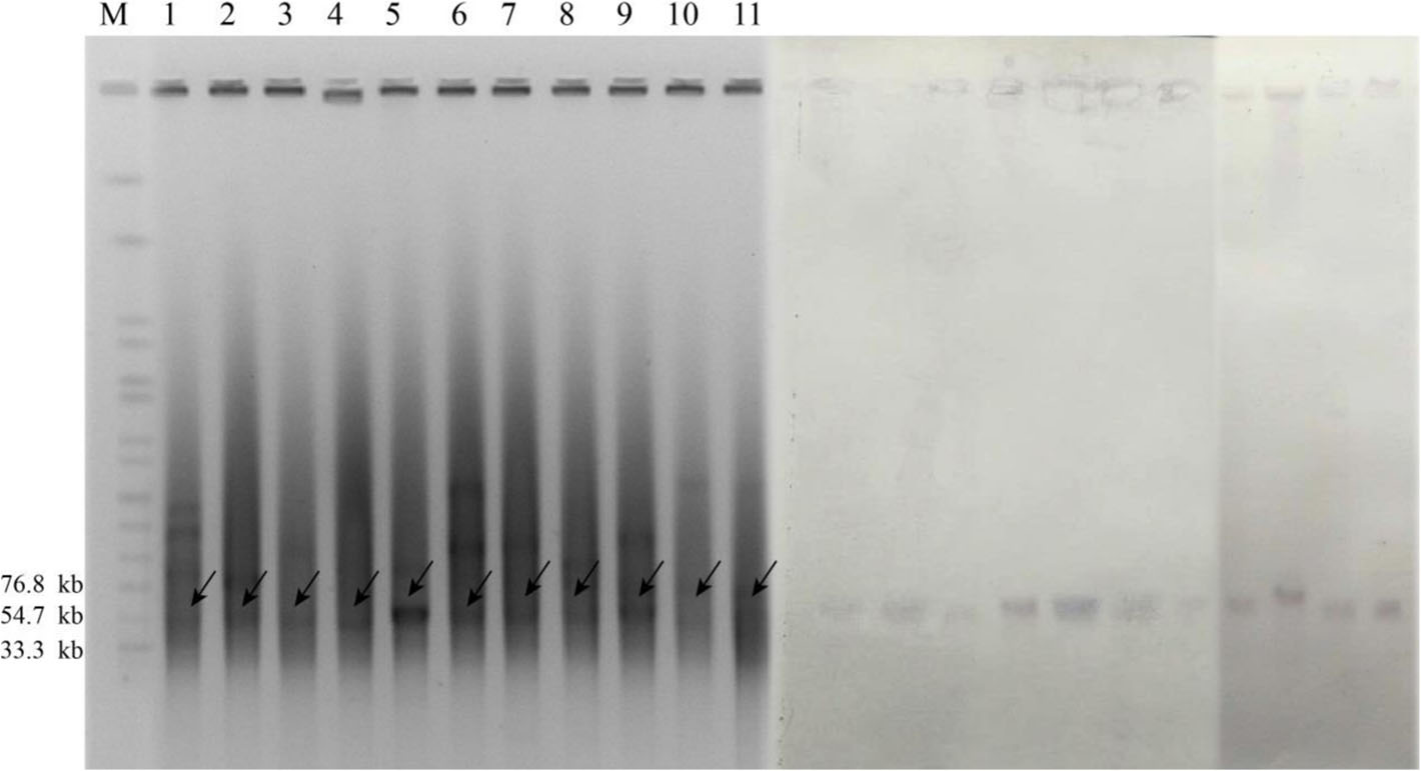
S1-digested plasmid DNA and southern blot hybridization of *bla*NDM-5 positive isolates. Bands in A with arrows pointing to them showed positive signals in Southern blot hybridization with the NDM-5 probe. M = *Salmonella serotype Braenderup* strain H9812 molecular marker. 1 = *K. pneumoniae* KP387; 2 = *E. coli* EC135; 3 = *E. coli* EC463; 4 = *E. coli* EC734; 5 = *E. coli* EC144; 6 = *E. coli* EC122; 7 = *E. coli* EC418; 8 = *C. freundii* CF418; 9 = *E. coli* EC310; 10 = *E. coli* EC611; 11 = *E. coli* EC126

Our recent studies showed that *bla*_NDM-5_ was able to coexist in the same isolate with tigecycline and colistin resistance phenotypes, thereby generating strains that approached pan-resistance. For example, *bla*_NDM-5_ was not only identified in high-level tigecycline resistance *E. coli* strains [21], but also coexisted in the same strain with the transferrable colistin resistance gene *mcr-1* [15]. It is clear that generating strains results in so-called “superbug” isolates and accelerating entery into a “postantibiotic” era [22].

### Genetic relatedness

MLST and PFGE experiments were performed to analyse the clonal relatedness of *bla*_NDM-5_-positive isolates because NDM-5 producers are infrequently isolated worldwide. According to the MLST results, nine *bla*_NDM-5_-postive *E. coli* isolates were grouped into 9 different sequence types. In accordance with the MLST results (Fig. 1), the different PFGE patterns confirmed that the seven *E. coli* isolates are not clonally related to each other even though some of the strains were collected from the same hospital. Strains EC122 and EC144 own similar the PFGE profiles, but the two strains have different sequence type and different resistance genes. Furthermore, core genome multi-locus sequence typing (cg-MLST) analysis in our study showed the *bla*_NDM-5_-positive isolates were not clonal relatedness (Additional file 1: Figure S1). In addition, the *K. pneumoniae* KP487 isolate belongs to ST182.

A previous study collected 11 NDM-5-producing *E. coli* strains from 7 hospitals in various locations in China from 2013 to 2014, and found that ST16*7 E. coli* strains in clinical settings exhibited close linkages with the *bla*_NDM-5_ gene [23]. Our previous study also showed that high-level tigecycline resistance *E. coli* strains carrying *bla*_NDM-5_ also belonged to the ST167 clonal lineage [21], indicating that the ST167 sequence type is an important reservoir of *bla*_NDM-5_ in China. However, the diversity of MLST and PFGE types in the present study showed that the *bla*_NDM-5_ gene has been carried in other STs *E. coli* isolates from 2016 to 2017. Moreover, the *bla*_NDM-5_ gene was detected in the *K. pneumoniae* and one *C. freundii* strains, indicating that this gene has further disseminated in *Enterobacteriaceae*. Note that NDM-5-related outbreak has been reported [24, 25]. Although no genetic association was found between our *bla*_NDM-5_-positive isolates with other strains, the widespread dissemination of *bla*_NDM-5_ in recent years in *Enterobacteriaceae* highlights the need for extensive attention.

### Location of the *bla*_NDM-5_ gene

S1-PFGE followed by Southern blot demonstrated that the *bla*_NDM-5_-positive strains were all located on plasmids of the same size(~ 46 Kb) (Fig. 2). The filter mating experiments were carried out to confirm the transferability of these *bla*_NDM-5_ plasmids. Nine of the 11 isolates tested could successfully transfer their carbapenem-resistant phenotype to *E. coli* strain C600 (Table 2). In addition,incompatibility plasmid classification showed that all the *bla*_NDM-5_ plasmids belonged to the IncX3-type plasmid. IncX3 plasmids might have played an important role in mediating the horizontal transmission of the *bla*_NDM_ gene. This possibility has been supported by the results of several studies [6, 26–29]. In this study, *bla*_NDM-5_ was carried by the IncX3 plasmids. Moreover, 81.8% (9/11) of isolates carrying this type plasmid were able to transfer carbapenem-resistant phenotype. However, conjugation experiments of *E. coli* EC126 and EC135 strains were not performed because these two strains were resistant to rifampin. To date, IncX3 plasmids carrying *bla*_NDM-5_ have been reported worldwide [3, 22, 23]. Therefore, our present study further supplements those previous studies. In addition, we isolated *E. coli* and *C. freundii* strains carrying *bla*_NDM-5_ from a single patient. These *bla*_NDM-5_-carrying plasmids had very similar sequences (99% coverage and 98% similarity), indicating probable horizontal transfer of *bla*_NDM-5_ between *E. coli* and *C. freundii* strains by one same plasmid. In addition, the plasmid stability experiments showed that the *bla*_NDM-5_-positive plasmids were all stable in these isolates. After 12 rounds of subculture in MH agar without antibiotic addition, the randomly selected strains all carried the *bla*_NDM-5_ gene and a plasmid identical to their parental isolate in size. Overall, it is important for the IncX3 type plasmid to play an important role in the further dissemination of *bla*_NDM-5_ in *Enterobacteriaceae.* Therefore, it is imperative that effective measures be taken immediately to control the spread of this plasmid.

**Table 2:** Antibiotic susceptibility of NMD5-producing isolates and their transconjugants

Isolates	MICs (mg/L)										
	FEP	IPM	NIT	CAZ	AMK	CIP	ATM	TGC	CPS2/1	MNO	COL
EC126	> 128	8	128	> 128	> 128	128	> 128	0.5	> 256	8	0.5
EC135	64	16	64	> 128	128	128	0.125	2	> 256	32	0.5
KP387	64	16	128	> 128	1	2	0.25	4	> 256	32	0.5
JH387	64	16	16	> 128	0.5	0.5	0.25	0.5	> 256	4	0.5
EC463	> 128	64	8	> 128	1	64	32	2	> 256	64	0.5
JH 463	128	64	16	> 128	1	0.125	0.125	0.25	> 256	2	< 0.25
EC734	64	8	8	> 128	1	64	4	0.25	> 256	32	0.5
JH734	64	16	16	> 128	0.5	0.25	0.125	0.5	> 256	2	< 0.25
EC611	32	8	8	> 128	1	0.0625	0.0625	0.25	> 256	2	0.25
JH611	64	8	8	> 128	0.5	0.0625	0.125	0.5	> 256	2	0.25
EC144	128	32	32	> 128	> 128	64	128	0.25	> 256	32	0.5
JH144	128	16	32	> 128	0.5	0.5	0.125	0.5	> 256	2	< 0.25
EC122	> 128	32	64	> 128	> 128	64	> 256	8	> 256	128	0.5
JH122	128	16	16	> 128	0.5	0.5	0.125	0.5	> 256	2	< 0.25
EC418	32	8	32	> 128	1	0.25	0.125	1	> 256	48	0.5
JH418	32	8	16	> 128	0.5	0.25	0.125	0.5	> 256	2	< 0.25
CF418	32	32	8	> 128	1	0.25	0.l25	0.5	> 256	4	0.5
JHF418	16	8	8	> 128	1	0.25	0.125	0.5	> 256	2	< 0.25
EC310	> 128	128	8	> 128	1	8	0.19	0.5	> 256	2	0.5
JHE310	> 128	64	8	> 128	0.5	0.5	0.125	0.5	> 256	1	< 0.25
EC600	0.125	0.5	8	0.25	0.5	0.125	0.25	0.125	0.5	1	< 0.25
ATCC25922^a^	0.125	0.5	< 8	0.125	0.5	0.125	0.125	0.125	0.25	0.25	< 0.25

*FEP* cefepime, *IMP* imipenem, *NIT* nitrofurantoin, *CAZ* ceftazidime, *AMK* amikacin, *CIP* ciprofloxacin, *ATM* aztreonam, *TGC* tigecycline, *MNO* minocycline, *CPS* cefperazone/sulbactam, *COL* colistin

All susceptibility tests were repeated at least three times according to CLSI method. The results of colistin susceptibility were interpreted according to EUCAST breakpoints

^a^quality control strain

### Plasmid sequence analysis of *bla*_NDM-5_

**Fig. 3:**
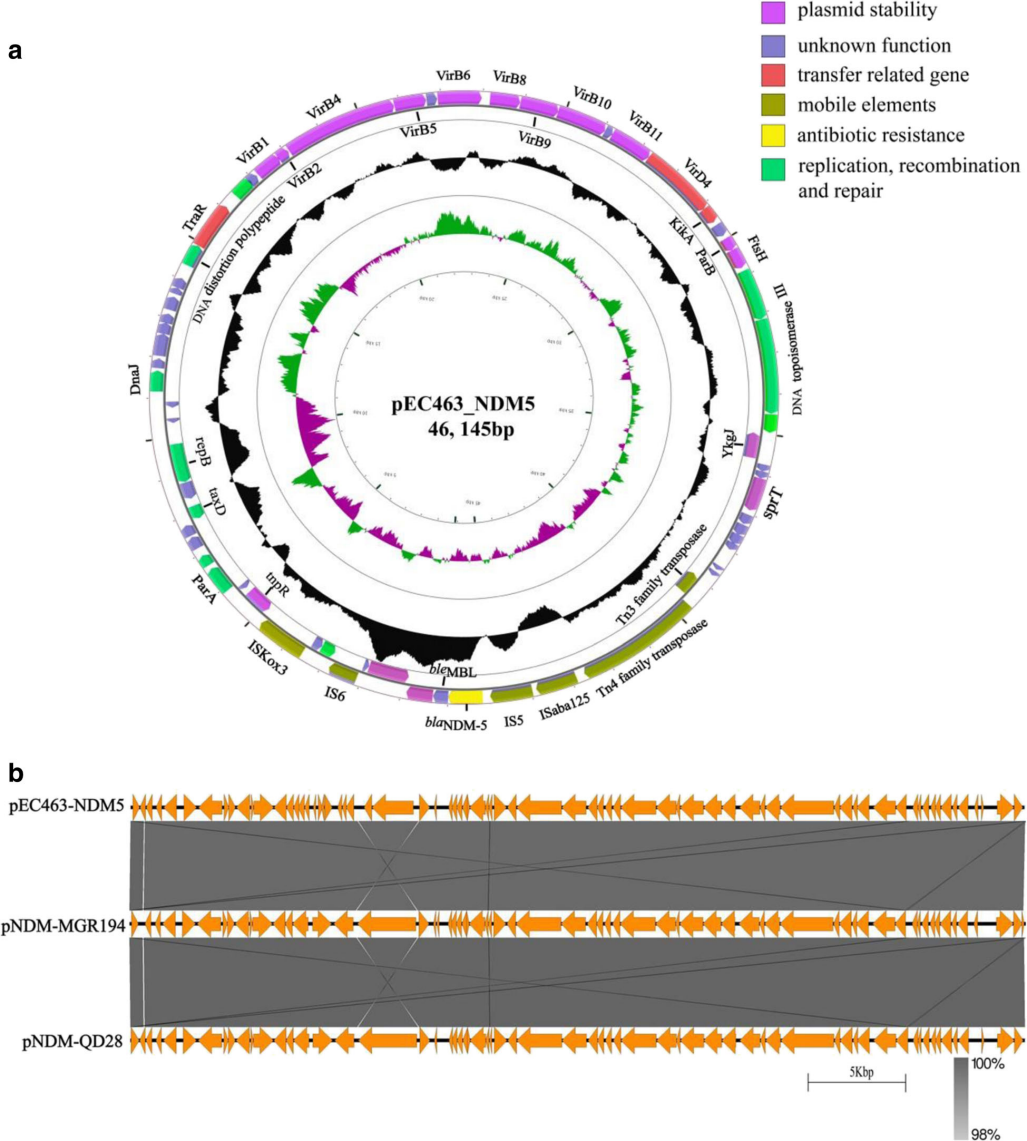
Plasmid analysis of pEC463-NDM5. Schematic map of plasmid p pEC463-NDM5 (**a**), comparative analysis of three *bla*NDM-5-carrying IncX3 plasmids (**b**). The putative open reading frames are shown as arrowheads orrods (less than 130 amino acids). The gene name is shown near the corresponding arrowhead or rod. The depthof shading is indicative of the percentage BLASTN match, as indicated on the bottom

The entire plasmid sequence was obtained to better characterize the *bla*_NDM-5_-positive plasmid. Sequence analysis showed that the plasmid was 46,145 bp in length (Fig. 3a). The *bla*_NDM-5_ gene was preceded by IS*3000*, IS*Aba125* and IS*5*, and followed by *ble*_MBL_, *trpF*, *dsbC*, IS*6* and IS*kox3*.No other antimicrobial resistance genes were detected in this plasmid.

Further sequence alignments based on BLAST revealed that the plasmid sequences showed almost identical nucleotide sequences with those of the previously reported IncX3 plasmids pNDM-MGR194 of *K. pneumoniae* MGR-K194 in India [8]. The plasmid pNDM-MGR194 carrying *bla*_NDM-5_ was reported in 2015 in India, which was considered to play an important role in the dissemination of the *bla*_NDM-5_ gene because pNDM-MGR194-like plasmid was highly similar to those plasmids reported in China [3], Australia [5] and Denmark [7]. In addition, most of the *bla*_NDM-5_-carrying plasmids reported in China belonged to the IncX3-type and were identical or near-identical to pNDM-MGR194-like plasmid (Table 3). In this study, identification of the IncX3-type pNDM-MGR194-like plasmid in *E. coli* of different STs, *K. pneumoniae* and *C. freundii* strains indicated that this plasmid could mediate inter- and intra-species transfer of *bla*_NDM-5_. This possibility was further supported by our conjunction experimental data in vitro. Moreover, this plasmid carried in *E. coli* and *C. freundii* strains was isolated from faeces sample of a single patient at the same time, providing strong evidence that this plasmid could mediate *bla*_NDM-5_ dissemination in *Enterobacteriaceae*. Overall, our results revealed that IncX3-type pNDM-MGR194-like plasmids facilitate the rapid dissemination of *bla*_NDM-5_ among *Enterobacteriaceae* in China.

**Table 3:** Detailed information of the *bla*_NDM-5_-habouring plasmids reported in the NCBI database

Inc. group	Transferability^a^	Size (kb)	Host strain	MLST	Sample	Country	Reference
IncX3	T	46^b^	*K. pneumoniae*	–	Human Blood	India	[8]
	–	46^b^	*E. coli*	ST1284	Human Groin	Denmark	[24]
	–	46^b^	*E. coli*	ST648	Human Urine	India	[5]
	C	46^b^	*E. coli*	ST167	Human Rectum	China	[6]
	C	46^b^	*E. coli*	ST167	Human Urine	China	[30]
	C	46^b^	*E. coli*	ST167	Human Blood	China	[30]
	C	46^b^	*E. coli*	ST2608	Human Swab	China	[30]
	C	46^b^	*E. coli*	ST5131	Human Vaginal secretions	China	[30]
	T	46^b^	*E. coli*	ST167	Human sputum	China	[3]
	T	46^b^	*E. coli*	ST167	Human Urine	China	[3]
	T	46^b^	*E. coli*	ST167	Human Blood	China	[21]
	T	46^b^	*E. coli*	ST167	Human Blood	China	[15]
	T	46^b^	*E. coli*	ST206	Human stool	China	[31]
	C	46^b^	*K. michiganensis*	–	Human stool	China	[32]
	C	46^b^	*E. coli*	ST446	Cows fecal	China	[11]
	C	46^b^	*E. coli*	ST2	Cows fecal	China	[11]
	C	46^b^	*E. coli*	ST3	Cows fecal	China	[11]
	C	46^b^	*E. coli*	ST354	Human ascites	China	this study
	C	46^b^	*E. coli*	ST746	Human feces	China	this study
	C	46^b^	*E. coli*	ST6395	Human blood	China	this study
	C	46^b^	*E. coli*	ST6335	Human pus	China	this study
	C	46b	*E. coli*	ST12	Human ascites	China	this study
	–	46^b^	*E. coli*	ST410	Human sputum	China	this study
	C	46^b^	*E. coli*	ST361	Human blood	China	this study
	C	46^b^	*E. coli*	ST167	Human urine	China	this study
	–	46^b^	*E. coli*	ST617	Human Urine	China	this study
	C	46^b^	*K. pneumoniae*		Human blood	China	this study
	C	46^b^	*C. freundii*	–	Human feces	China	this study
IncF	–	> 100	*E. coli*	ST648	Human throat	UK	[2]
	T	> 100	*E. coli*	–	Human pus	India	[33]
	T	> 100	*E. coli*	–	Human pus	India	[33]
IncFII	T	84.5	*Salmonella enterica serovar Typhimurium*	ST34	Human fecal	China	[34]
	C	110	*E. coli*	ST418	Human stool	Poland	[35]
	C	90	*E. coli*	ST418	Human urine	Spain	[36]
IncN	C	110	*E. coli*	ST540	Human feces	Japan	[37]
Untypeable	C	48	*K. pneumoniae*	ST231	Human urine	Singapore	[38]

^a^C: plasmid is able to transfer to *E. coli* recipients by conjugation; T: plasmid is able to transfer to *E. coli* recipients by transformation or electroporation

^b^These plasmids are identical or near-identical to plasmid pNDM-MGR194

## Conclusions

We report a near-term epidemiological study demonstrating the further dissemination of *Enterobacteriaceae* with the *bla*_NDM-5_ gene in China. Our work provides evidence that the IncX3-type plasmid played an important role in the dissemination of *bla*_NDM-5_ in *Enterobacteriaceae*. In addition, to the best of our knowledge, this report is the first to isolate *E. coli* and *C. freundii* strains carrying *bla*_NDM-5_ from a single patient. Close surveillance is urgently needed to monitor the further spread of NDM-5-producing isolates.

## Additional file

Additional file 1: cg-MLST of blaNDM-5-positive isolates. (DOCX 61 kb)

## Abbreviations

cg-MLST: Core genome multi-locus sequence typing
CLSI: Clinical & Laboratory Standards Institute
CRE: Carbapenem-resistant *Enterobacteriaceae* isolates
MIC: Minimum inhibitory concentration
MLST: Multilocus sequence typing
NDM: New Delhi metallo-β-lactamase
PFGE: Pulsed-field gel electrophoresis
RAST: Rapid Annotation using Subsystems Technology
