# Supplementary material for: Dissemination of blaNDM-5 gene via an IncX3-type plasmid among non-clonal Escherichia coli in China
Source: Antimicrob Resist Infect Control. 2018 Apr 26;7:59. doi: 10.1186/s13756-018-0349-6 (PMC5918551; doi:10.1186/s13756-018-0349-6)
Supplement: Supplementary file 1 — cg-MLST of blaNDM-5-positive isolates. (DOCX 61 kb) [file 13756_2018_349_MOESM1_ESM.docx]

**1. cgMLST profile**

Genome-wide gene-by-gene comparison approach was used to determine the genetic relatedness using SeqSphere+ version 4.1.9 (Ridom GmbH, Münster, Germany). The thresholds for interpreting clonality with cgMSLT were as follows: <35 allelic differences are considered related, 35 or more allelic differences are considered unrelated ^[1]^.


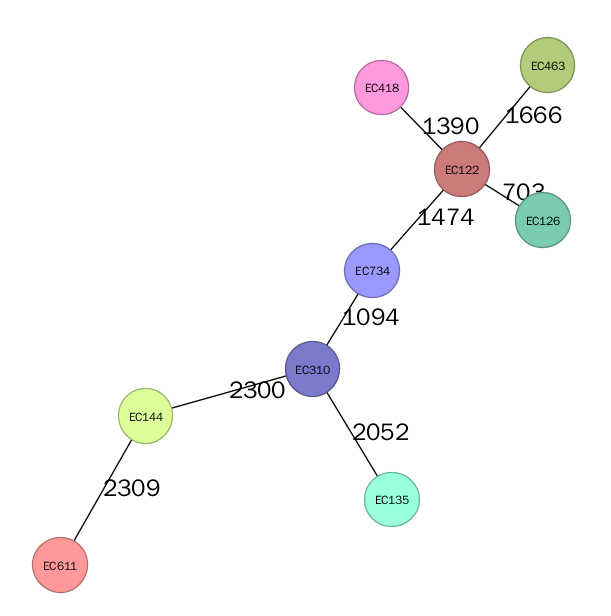


FIG.S1 Minimum spanning tree of *E. coli* isolates producing NDM-5. Distance based on a cgMLST of 2411 genes using the parameters “pairwise ignoring missing values” during calculation. Number of different alleles is indicated on the edges between connected isolates (nodes). Isolates are presented by their ID.

[1]. Xuewei Zhou,1,*† Silvia García-Cobos,1,*† Gijs J. H. M. Ruijs, et al. Epidemiology of Extended-Spectrum β-Lactamase-Producing *E. coli* and Vancomycin-Resistant *Enterococci* in the Northern Dutch–German Cross-Border Region. Front Microbiol. 2017; 8: 1914.
